# Integrin-linked kinase functions as a downstream signal of platelet-derived growth factor to regulate actin polymerization and vascular smooth muscle cell migration

**DOI:** 10.1186/1471-2121-11-16

**Published:** 2010-02-23

**Authors:** Mitra Esfandiarei, Sahar Abdoli Yazdi, Virginia Gray, Shoukat Dedhar, Cornelis van Breemen

**Affiliations:** 1 Child & Family Research Institute, Department of Anesthesiology, Pharmacology, and Therapeutics, University of British Columbia, 950 West 28th Avenue, Vancouver, BC, V5Z 4H4, Canada; 2 Department of Cancer Genetics, British Columbia Cancer Research Centre, University of British Columbia, 675 West 10th Avenue, Vancouver, BC, V5Z 1L3, Canada

## Abstract

**Background:**

Vascular smooth muscle cell migration and accumulation in response to growth factors extensively contribute to the development of intimal thickening within the vessel wall. Cumulative evidence has shown that actin cytoskeleton polymerization and rearrangement are critical steps during cellular spreading and migration. Integrin-linked kinase, an intracellular serine/threonine kinase, is a cytoplasmic interactor of integrin beta-1 and beta-3 receptors regulating cell-cell and/or cell-extracellular matrix interaction, cell contraction, extracellular matrix modification, and cell spreading and migration in response to various stimuli. However, the regulatory role of ILK during vascular smooth muscle cell migration and the importance of integrin signaling in occlusive vascular diseases are not yet fully elucidated.

**Results:**

In the present study, we report that integrin-linked kinase controls mouse aortic smooth muscle cell migration in response to platelet-derived growth factor. We have also identified p38 mitogen activated protein kinase as a downstream signaling pathway of the integrin-linked kinase that regulates platelet-derived growth factor-induced actin polymerization and smooth muscle cell migration.

**Conclusion:**

This study will provide new insights into the potential therapeutic value of modulating integrin signaling in an attempt to block or delay smooth muscle cell migration and the progression of vascular diseases.

## Background

The initiation and progression of intimal thickening in arterial walls is largely due to migration and subsequent proliferation of smooth muscle cells (SMCs) in the sub-intimal space in response to various stimuli including oxidized low density lipoprotein (oxLDL), circulating growth factors, and inflammatory cytokines such as platelet-derived growth factor (PDGF), tumor necrosis factor (TNF)-α, and interleukin-1 (IL-1) [1,2]. Of these, PDGF, a growth factor released by vascular SMC, endothelial cells, and platelets, has been reported as the most potent inducer of SMC migration within the injured area of the vascular wall.

PDGF is a heparin-binding growth factor composed of polypeptide chains that can be assembled into homodimers (PDGF-AA, -BB) or heterodimer (PDGF-AB) structures and bind to two related cell-surface receptors with tyrosine kinase activity, PDGF receptor α and β [3-5]. In the last few years two additional homodimers PDGF-CC and PDGF-DD were also discovered [6-9]. PDGF binding to its cognate receptors results in dimerization, and subsequent auto-phosphorylation of specific tyrosine residues outside the kinase domain, creating a docking site for SH2 domain-containing signaling proteins. A large number of SH2 domain containing proteins including the phsophatidylinositol-3 kinase (PI3K), phospholipase C-γ (PLC-γ), the tyrosine phosphatase SHP-2, Ras GTPase activating protein (Ras-GAP), Grb2, Grb7, Nck, and the Src family of tyrosine kinases have been shown to bind to cytoplasmic tails of PDGF receptors activating various downstream signaling proteins involved in cell growth, proliferation, survival, and migration [10].

Cellular migration is regulated via a complex interaction between growth factors that attach to their cognate receptors, transmembrane integrins that bind to the components of extracellular matrix, and mechanical stress, all of which cooperatively induce polymerization and reorganization of the actin cytoskeleton. These events are coordinated by mechanisms involved in assembly or disassembly of local adhesion sites, transient changes in actin filaments dynamics, and formation of discrete structures such as stress actin fibers, membrane ruffles, lamellipodia, and filopodia [11-13]. Integrin-linked kinase (ILK), a serine-threonine protein kinase containing a catalytic domain at C terminus, a central pleckstrin homology (PH)-like domain, and four ankyrin-like repeats at the N terminus, is an important component of focal adhesion complex anchoring actin filaments to integrin receptors and the cell membrane [14]. Previous studies have demonstrated that ILK regulates fibroblast migration through the phosphatidylinositol-3 kinase (PI3K) [15], osteosarcoma cell spreading and motility via Rho-associated kinase (ROCK) [16], and mammary epithelial cell migration through the guanine nucleotide exchange factor α-PIX [17].

There is also growing evidence on the cooperation between PDGF receptor and integrins in regulating cellular survival and adhesion [18-20]. However, little is known about the existence of cross-talk between ILK and PDGF signaling in vascular smooth muscle cells and the probable regulatory role of ILK during PDGF-induced SMC migration. In the present study, we have examined the potential function for ILK as an upstream protein regulating the migratory response to PDGF in a primary mouse aortic SMC culture. We have also characterized one downstream pathway that mediates the regulatory role of ILK in modulation actin polymerization and SMC migration.

## Results

### PDGF activates integrin-linked kinase in mouse aortic SMCs

**Figure 1:**
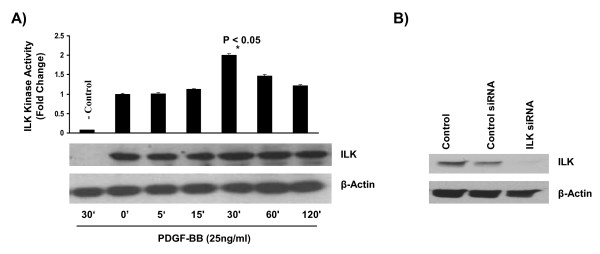
**PDGF treatment increases integrin-linked kinase activity in mouse aortic smooth muscle cell culture**. **A) **Primary mouse aortic SMCs were serum-starved overnight and then treated with 25 ng/ml of PDGF-BB. ILK kinase activity was measured at various timepoints following treatment. PDGF-BB increased ILK kinase activity (two fold increase). SMC culture transfected with ILK siRNA was used as negative control in the assay. As shown in first lane of the blot, ILK expression was completely abolished in negative control group that led to a significant decrease in ILK kinase activity in this group. **B) **Transfection of mouse aortic SMCs with ILK siRNA for 96 hours completely blocked ILK protein expression. Data presents three independent experiments and is presented as mean ± STDEV (n = 3), where *P *< 0.05 was considered significant.

To study the effect of PDGF treatment on ILK activation, mouse aortic SMCs were treated with 25 ng/ml of PDGF-BB for various timepoints and cell lysates were collected and subjected to the kinase activity assay. As shown, PDGF treatment increased ILK kinase activity around 30 minutes post treatment in the SMC culture without having any significant effect on total ILK protein expression. PDGF-induced ILK kinase activity declined around 60 minutes after treatment (figure 1A). To ensure the specificity of the test and as a negative control, a group of SMCs culture was transfected with specific ILK siRNA for 96 hours. Such treatment completely abolished ILK protein expression in SMCs (figure 1B). As shown, very little kinase activity was detected in ILK-siRNA group confirming that the results of the kinase activity assay is specific. It is noteworthy that we carefully monitored the morphology of SMCs transfected with ILK siRNA for the incubation period prior to PDGF treatment (96 hours) to assure that siRNA treatment had no cytotoxic effect on mouse SMC (data not shown).

### PDGF induces SMC migration through an ILK-dependent pathway

**Figure 2:**
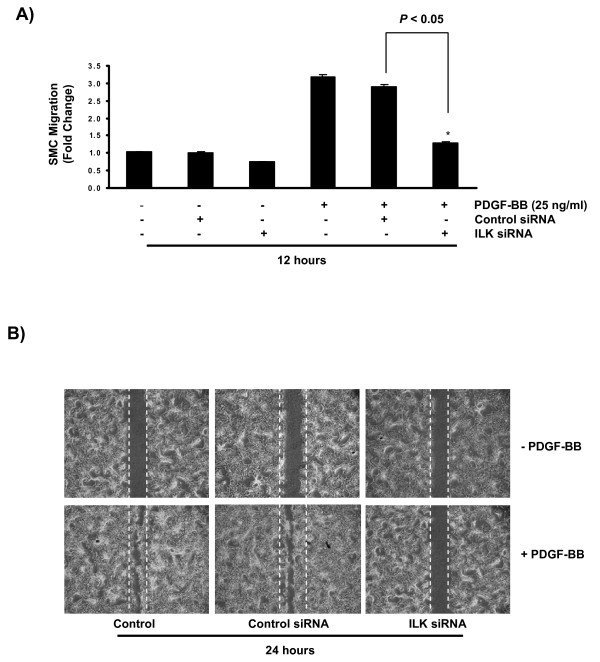
**PDGF induces SMC migration through anILK-dependent pathway**. **A) **Inhibition of ILK protein expression with 20 nM ILK siRNA markedly decreased SMC migration in response to PDGF-BB. Data represents three independent experiments (n = 3 culture plates in each replicate) and the value is presented as mean ± STDEV where *P *< 0.05 was considered significant. **B) **ILK inhibition decreased the number of migrated SMCs into the site of injury in response to PDGF-BB treatment as compared to the control group (contorl siRNA). Original magnification, ×40. Data present one of three independent experiments.

To investigate the potential function of ILK in regulation of PDGF-induced SMC migration, cells were transfected with specific ILK siRNA, and then treated with 25 ng/ml of PDGF-BB. The migratory response was measured using both modified Transwell Boyden chamber and wound healing assays. Inhibition of ILK protein expression markedly decreased SMC migration indicating that the presence of ILK was required for transducing the migratory signal stimulated by PDGF-BB (figures 2A &2B).

PDGF is considered as a very potent mitogen. The observed effect of PDGF-BB on wound closure in figure 2B could be due to the effect of this growth factor on SMC proliferation and/or migration. Thus, to determine whether the proliferatory effect of PDGF-BB had contributed to the observed increase in cell migration., cells were treated with increasing doses of PDGF-BB for 24 hours (the maximum time required for wound closure), and cell proliferation and migration were measured using the modified Transwell Boyden chamber assay and MTS cell proliferation assay, respectively. Treatment of primary mouse SMCs with 25 ng/ml of PDGF-BB significantly increased cell migration without having any noticeable effect on SMC proliferation. To assure the accuracy of both assays and as a positive control, mouse SMC culture were also treated with 10% serum. These findings emphasized that the observed increase in cell migration in both assays used in this study (24 hours post injury or following loading onto Transwell Boyden Chambers) was due to the pro-migratory, not proliferatory, effect of PDGF-BB on mouse aortic SMC (additional file 1, *figure S1*).

### ILK inhibition abolishes PDGF-induced p38 MAPK activation in mouse aortic SMC culture

Studies in various *in vitro *models, have shown that members of the family of the mitogen activated protein kinases (MAPKs) become activated and play a crucial role in regulating cell migration in response to PDGF treatment [21-24]. However, the actual mechanisms underlying this activation and the identity of the upstream kinases involved are not fully elucidated. Here, we first characterized the dynamics of MAPKs family activation in response to PDGF-BB treatment in our primary mouse aortic SMC model. In agreement with previous reports in various cell models, PDGF-BB treatment resulted in the phosphorylation of all three members of MAPKs family, extracellular signal-regulated protein kinase 1/2 (Erk1/2), Jun N-terminus kinase (JNK), and p38 MAPK starting around 5 minutes and with a peak activation at 20-30 minutes post-treatment (additional file 2, *figure S2*).

**Figure 3:**
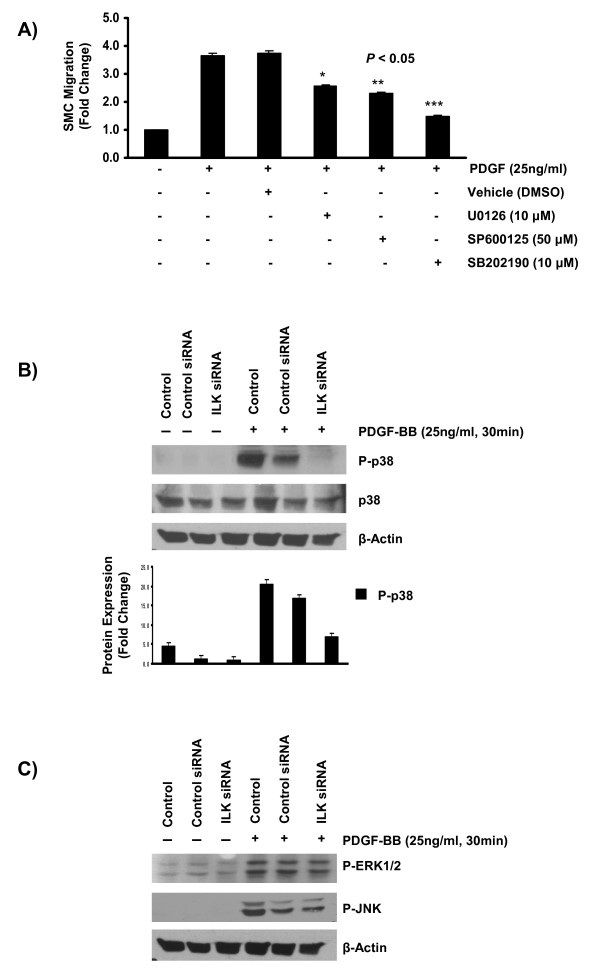
**ILK inhibition abolishes PDGF-induced p38 MAPK activation in mouse aortic SMCs**. **A) **Mouse aortic SMCs were treated with specific MAPKs inhibitors at specified concentrations for 2 hours prior to seeding onto Transwell Boyden Chambers and PDGF-BB treatment (25 ng/ml) and during the incubation period (12 hours), and cell migration was measured at 12 hours post culture. As shown, inhibition of all three members of MAPKs family significantly reduced SMC migration in response to PDGF-BB. Data is the representative of three independent experiments (n = 3 cell culture replicates for each experiments). **B) **Mouse aortic SMCs were transfected with 20 nM of specific ILK siRNA and then treated with 25 ng/ml of PDGF-BB for specified timepoints. Inhibition of ILK protein expression markedly reduced PDGF-induced p38 MAPK phosphorylation in mouse aortic SMCs. The blot presents one of three independent experiments. The value in the graph is presented as the mean ± STDEV of three independent experiments. **C) **ILK inhibition by specific siRNA had no detectable effect on Erk1/2 and JNK phosphorylation in response to PDGF-BB treatment in mouse aortic SMC culture. Data presents one of three independent experiments (n = 3 cell cultures for each independent experiments).

To investigate the role of each member of MAPKs family in regulating PDGF-induced cell migration, SMCs were treated with specific MEK/Erk1/2 inhibitor (20 μM U0126), JNK inhibitor (50 μM SP600125), and p38 MAPK inhibitor (10 μM SB202190) for two hours prior to PDGF-BB treatment and cell migration was measured using the modified Transwell Boyden Chamber assay. Inhibition of all members of MAPKs family markedly reduced mouse SMC migration in response to PDGF-BB treatment (figure 3A).

Various components of focal adhesion complex have been shown to regulate or cross talk with members of MAPKs family [24]. ILK, as a key constituent of the focal adhesion complex, also plays an important function in transducing inside-out and outside-in signals [25]. To investigate the role of ILK in regulating MAPKs activation in response to PDGF, SMCs were transfected with specific ILK siRNA prior to PDGF-BB treatment. Inhibition of ILK expression with specific siRNA resulted in significant decrease in PDGF-induced p38 MAPK phosphorylation (figure 3B), while having no effect on Erk1/2 and JNK phosphorylation (figure 3C). This observation suggests that ILK presence is required for PDGF-induced phosphorylation of p38 MAPK in mouse SMC.

### ILK mediates PDGF-induced mouse aortic SMC migration through a p38 MAPK-dependent pathway

Taking into account that PDGF-BB activates both ILK and p38 MAPK in mouse aortic SMCs (figures 1A & Additional file 1, figure S1), and the observation that p38 MAPK has a crucial role in PDGF-induced cell migration (Figure 3A); we sought to determine whether ILK can also modulate the migratory effect of p38 MAPK activation in response to PDGF-BB. To examine this hypothesis, we utilized a gain and loss of function approach using adenoviral constructs of ILK and/or p38 MAPK.

**Figure 4:**
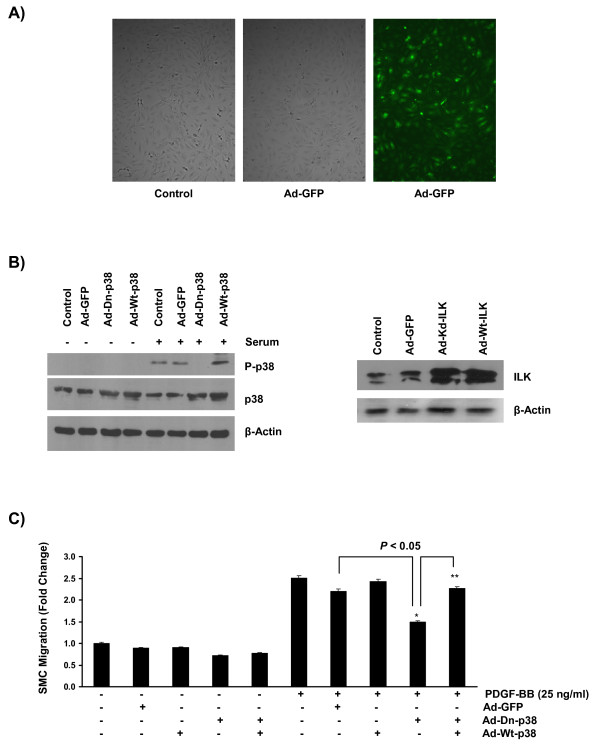
**Dominant negative form of p38 MAPK significantly decreases SMC migration in response to PDGF**. **A) **Photomicrograph of mouse aortic SMCs showing the morphology and GFP expression at 36 hours following transfection. Sub-confluent SMCs were infected with adenoviral vector at the multiplicity of infection of 100. Note the high level of GFP expression as well as the normal phenotype of Ad-GFP-transfected SMCs. Original magnification, ×200. **B) **Over-expression of a dominant negative form of p38 MAPK markedly increased total p38 MAPK expression while blocking p38 MAPK phosphorylation in response to serum treatment in SMCs (serum is used as a strong activator for p38 MAPK). Also, note the considerable increase in ILK expression level in SMCs transfected with adenoviral ILK constructs. **C) **For migration assay, transfected SMCs were cultured in Transwell Boyden Chambers in the presence or absence of 25 ng/ml of PDGF-BB and cell migration was measured 12 hours post culture. Dominant negative p38 MAPK significantly decreases SMC migration in response to PDGF-BB treatment while expression of a wild type form of p38 MAPK increases SMC migration in response to PDGF-BB. In addition, over-expression of a wild type mutant of p38 MAPK subverted the inhibitory effect of p38 inhibition on SMC migration. Data represents three independent experiments (n = 3 cell cultures for each independent experiments).

In order to revalidate the important role of p38 MAPK activation during the migratory response, and also to exploit a more target-specific approach, we first infected primary mouse SMCs with adenoviral constructs expressing either a dominant negative form of p38 MAPK (Ad-Dn-p38), a wild type form of p38 MAPK (Ad-Wt-p38), or a GFP (Ad-GFP) protein. Western blot analysis (measuring protein expression) and fluorescent microscopy (visualizing GFP expression) were used to evaluate the transfection efficiency at 36 hours post transfection (Figures 4A &4B).

In concurrence with our previous observations, over-expression of a dominant negative mutant of p38 MAPK significantly reduced PDGF-induced SMC migration (Figure 4C). Furthermore, over-expression of a wild type form of p38 rescued SMC migration in response to PDGF-BB, corroborating the specificity of p38 MAPK inhibition in mouse SMC culture transfected with the Ad-Dn-p38 MAPK construct (Figure 4C).

**Figure 5:**
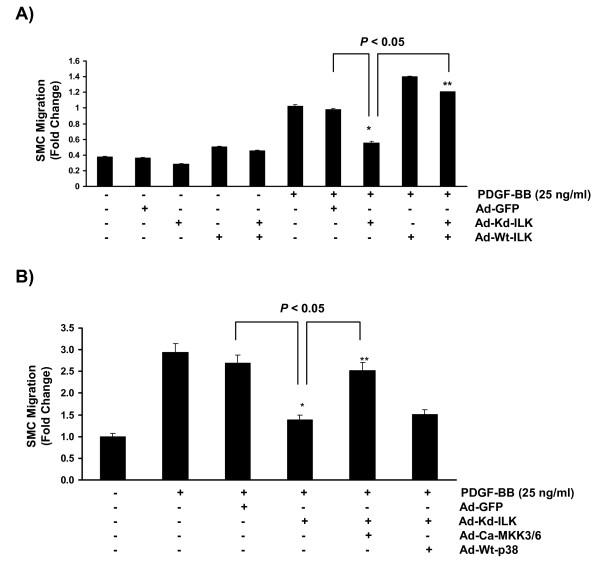
**ILK regulates PDGF-induced SMC migration through a p38 MAPK-dependent pathway**. **A) **Kinase deficient mutant of ILK significantly reduced PDGF-induced SMC migration in a Traswell Boyden Chamber migration assay. As shown, expression of a wild type form of ILK rescued SMC migratory response indicating the specificity of ILK inhibition by the adenoviral construct. **B) **Over-expression of an active form of MKK3/MKK6 (specific upstream activators of p38 MAPK), but not a wild type form of p38 MAPK, rescued SMC migration in response to PDGF-BB indicating that an active form of ILK is required for the initiation of a p38 MAPK-dependent migratory response to PDGF-BB in mouse aortic SMCs. (n = 3 cell cultures for each independent experiments).

Moreover, a kinase-deficient form of ILK (E359K), which is also incapable of binding to paxillin and parvin and therefore unable to participate in formation of the focal adhesion complex, resulted in a significant decline in PDGF-induced SMC migration, an event that can be reversed by a wild type form of ILK (Figure 5A).

Finally, to establish a cause and effect association between inhibition of ILK, a downstream decrease in p38 MAPK phosphorylation, and the subsequent decline in cell migration, we co-transfected cells with a kinase deficient form of ILK (E359K) along with an active form of MKK3/6 (the specific upstream activator of p38 MAPK) or a wild type form of p38 MAPK. As shown, expression of Ad-Ca-MKK3/6 significantly reversed the inhibitory effect of ILK inhibition (Ad-Kd-ILK) in PDGF-treated SMCs (Figure 5B). In contrast, over-expression of the wild type p38 MAPK in SMCs already transfected with Ad-Kd-ILK, did not rescue cell migration, confirming our previous observation that the existence of an active form of ILK was required to facilitate the p38 MAPK-mediated migratory response to PDGF-BB (Figure 5B).

### ILK and p38 MAPK regulate PDGF-induced actin cytoskeleton polymerization in vascular SMC

**Figure 6:**
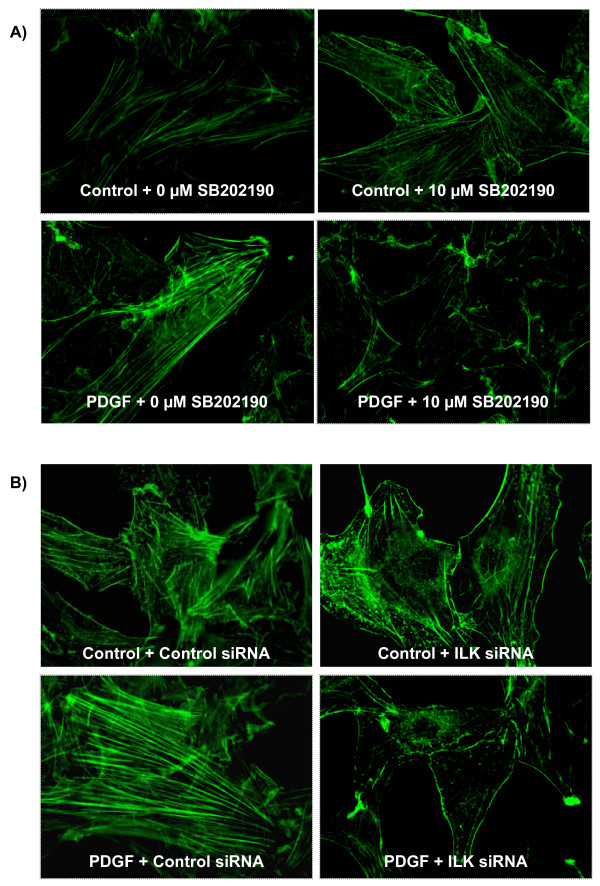
**ILK and p38 MAPK regulate PDGF-induced actin cytoskeleton polymerization in vascular SMCs**. **A) **Mouse SMCs were treated with either vehicle (DMSO) or with 10 μM of p38 MAPK specific inhibitor SB202190 for 1 hour prior to PDGF treatment. Following treatment, cells were washed and fixed and then stained for actin filaments. Inhibition of p38 MAPK markedly blocks PDGF-induced actin polymerization and reorganization (original magnification ×400). **B) **Mouse aortic SMCs were transfected with 20 nM of specific ILK siRNA and then treated with 25 ng/ml of PDGF-BB for 20 minutes. Cells were then fixed and stained for actin filaments. Inhibition of ILK expression in SMCs significantly reduced PDGF-induced actin polymerization and reorganization in mouse aortic SMCs. Images are representative image of three independent experiments (original magnification ×400).

Actin polymerization and re-organization is a critical step in cell motility and migration. To characterize the role of ILK and p38 MAPK in regulating actin polymerization in response to PDGF, cells were transfected with specific ILK siRNA (20 nM) or treated with specific p38 MAPK inhibitor (10 μM SB202190) prior to PDGF treatment. Inhibition of ILK expression and p38 MAPK activity significantly blocked PDGF-induced actin polymerization and re-organization in mouse SMC, indicating a crucial role for ILK and p38 MAPK in regulation actin-mediated cell migration (Figures 6A, B).

## Discussion

Vascular SMC migration contributes to the intimal thickening in occlusive vascular diseases. Following vascular injury, SMC migratory response to various stimuli begins with activation of cell surface receptors followed by remodeling events that involve focal adhesion complex and filament actin remodeling. Various cytokines, peptide growth factors, and components of the extracellular matrix (ECM) have been characterized as pro-migratory molecules.

PDGF is a very potent stimulant for SMC migration through activation of several signaling pathways [26-28]. In vascular SMC, PDGF activates multiple signaling cascades including the PI3K/Akt, protein kinase A (PKA), Src, Ras, and MAPKs signaling. The family of MAPKs comprises three distinct protein kinases, p38 MAPK, JNK, and Erk1/2, all of which have been shown to be involved in vascular diseases and remodeling [23].

On another note, integrin clustering and its downstream signals are necessary for SMC adhesion to ECM, an event that also initiates pro-migratory responses within the vascular wall. Mawatari and colleagues have shown that in human saphenous vein SMCs beta-3 integrin is required for growth factor and cytokine-induced proliferation [29]. It is believed that a coordinated cross-talk between integrins and growth factor tyrosine kinase receptors is necessary for the effective modulation of cell survival, growth, proliferation, and migration [30].

ILK is a key protein of focal adhesion complex connecting β1 and β3 integrins in plasma membrane to the actin cytoskeletal machinery. ILK also interacts with growth factor receptors through the NH2-terminal ankyrin repeat domain and small GTPase signaling molecules such as Nck-2 [14,31]. Previous reports on the effect of PDGF on ILK expression and activity in non-muscle cell cultures were controversial. While Campana et al reported on a PDGF-induced increase in ILK activity in primary rat Schwann cells [32], Janji and colleagues proposed that PDGF had no effect on ILK expression in melanoma HT-144 cells [33], indicating that the outcome of PDGF treatment on ILK signaling is cell specific.

In the present study, we have shown that PDGF-BB treatment results in an increase in ILK kinase activity in mouse aortic SMC culture. It is of importance that the increase in ILK kinase activity in SMCs is associated with a considerable increase in expression of both β1 and β3 integrins (Esfandiarei & van Breemen, unpublished observation). It is well known that the adhesive function of integrin receptors affects cytoskeleton rearrangement and cell motility. Therefore, it is presumable that increase in β1 and β3 integrins expression contributes to the pro-migratory effect of PDGF-BB in SMC culture. However, further investigation is required to evaluate the potential cross-talk between β1 and β3 integrins and PDGF receptors and the significance of such events in vascular SMC migration.

Numerous studies have provided a wealth of information on the crucial regulatory role for ILK as a protein kinase and also as a molecular scaffold and adaptor protein during cell spreading and migration in a variety of cancer cells [13,34]. Moreover, A recent study has shown that an increase in ILK expression coincides with higher rate of SMC migration within the atherosclerotic plaque in ApoE -/- mice, a reliable model that mimics the progression of atherosclerosis in humans [35]. On the contrary, Ho et al has shown that following balloon catheter injury of the rat carotid artery, a significant decrease in ILK expression is associated with an elevation in vascular SMC proliferation and migration [36]. Nevertheless, there is no clear understanding as to how ILK contributes to the intimal thickening in response to growth factors and cytokines released during the development of the atherosclerotic plaque, and the mechanisms by which ILK may modulate vascular SMC migration to the sub-intimal layer of the vascular wall.

In this study, we examined the role of ILK in cell migration, by inhibiting (using siRNA) or increasing (using an adenoviral vector expressing wild type ILK) ILK expression. Inhibition of ILK by different means significantly decreased cell migration in response to PDGF-BB, demonstrating that ILK plays an important role in regulating PDGF-induced vascular SMC migration. Expression of a wild type form of ILK increased SMC migration even in the absence of growth factor. It is noteworthy that the effect of wild type ILK in increasing SMC migration is more evident when SMCs were treated with PDGF-BB (figure 6A). These observations are in agreement with previous report by Dwivedi et al [37] that over-expression of a dominant negative form of ILK markedly reduced intimal thickening in human saphenous vein organ culture.

ILK mutation within the catalytic domain (E359K) disrupts the interaction and binding of ILK with two important components of focal adhesion complex, β-parvin and paxillin. This interaction is required for the assembly of a functional focal adhesion complex and subsequent actin polymerization and reorganization [38,39]. Over-expression of the E395K mutant of ILK resulted in significant reduction in PDGF-induced SMC migration. The effect was fully reversed by a wild type form of ILK. These findings further underscore the crucial function of ILK as molecular scaffold during SMC migration.

In an effort to understand how ILK mediates the pro-migratory effect of PDGF-BB in primary mouse SMC using RNA silencing technique, we were able to show that ILK inhibition could lead to a considerable decrease in p38 MAPK phosphorylation, while having no visible effect on Erk1/2 and JNK phosphorylation in response to PDGF-BB treatment. We also demonstrated that over-expression of an active form of MKK3/6 (specific upstream activator of p38 MAPK) dramatically reversed the inhibitory effect of ILK inhibition on SMC migration. Expression of a wild type form of p38 MAPK markedly increased SMC migration in the presence of PDGF-BB; however, it could not restore migratory response in cells over-expressing a kinase deficient form of ILK. It is of importance that the latter observation accentuates the view that the presence of a functional ILK is required for the initiation of a p38 MAPK-dependent migratory response to PDGF-BB in mouse aortic SMCs. Further studies are required to characterize the precise mechanisms underlying the ILK-mediated regulation of p38 MAPK in SMCs.

Actin polymerization and reorganization is a crucial step in cell migration. We have shown that PDGF increases actin polymerization and filament reorganization in SMCs. This event was significantly blocked by both ILK siRNA and p38 MAPK inhibitor, confirming the importance of this pathway in regulating SMC migration. The actual mechanism by which p38 MAPK may regulate cell migration is not well understood. Actin polymerization and reorganization is under rigorous regulation by a group of protein kinases and/or phosphatases that coordinate the constant turnover of actin filaments in a stimulated cell. Among these, cofilin is a conserved actin-binding protein that enhances actin filament reorganization through de-polymerization and severing of preexisting actin filaments [40]. It is well known that phosphorylation of cofilin (serine 3) by LIM kinase (LIMK) and testicular protein kinase 2 (TESK-2) results in deactivation of cofilin and subsequent inhibition of actin reorganization [41,42]. In mouse aortic SMCs, PDGF treatment resulted in rapid de-phosphorylation and activation of cofilin around 5-15 minutes post-treatment (additional file 3, figure S3). PDGF also increased LIMK phosphorylation leading to phosphorylation and deactivation of cofilin (additional file 3, figure S3). The feedback mechanism provides a strict control system for cell responses to migratory stimuli such as PDGF. Our preliminary observation suggests that p38 MAPK may control actin reorganization partly via regulating the de-phosphorylation and consequent activation of cofilin (Esfandiarei & van Breemen, unpublished observation). Further detailed studies are ongoing in our laboratory to characterize the signaling network and downstream pathways mediating such regulatory events.

## Conclusion

In summary, we have provided evidence that ILK is an important regulator of mouse vascular smooth muscle cell migration in response to PDGF-BB by modulating p38 MAPK phosphorylation. To our knowledge, this is the first report of the regulation of p38 MAPK by integrin signaling in mediating platelet-derived growth factor-induced migration in vascular smooth muscles. Future in vivo studies using specific small molecule inhibitors for ILK and/or p38 MAPK as well as developing transgenic animal models will provide additional information on the potential therapeutic value of integrin signaling, in general, and the integrin-linked kinase, in particular, in controlling vascular smooth muscle cell migration during the progression of occlusive vascular diseases.

## Methods

### Preparation of Primary SMC Culture

All surgical procedures and animal care were conducted according to the Guidelines for Animal Experiments of Faculty of Medicine, University of British Columbia approved by the University Committee on Ethics of Animal Experiments. Primary mouse aortic SMCs were isolated as described previously [43]. Breifly, aortic segments were isolated, cleaned of excess advential tissue, and digested using collagenase II (0.5 mg/ml). Isolated cells were pelleted, resuspended, and grown in Dulbecco's modified Eagle's medium. Sub-confluent (60-80%) cutlures were used for all experiments. ILK recombinant adenoviral constructs were kind gifts from Dr. Hyo-Soo Kim (Seoul National University Hospital, Korea). Adenoviral constructs for recombinant p38 MAPK and constitutively active forms of MKK3/6 were kindly provided by Dr. Donald R. Menick (Medical University of South Carolina, USA).

### Transient Transfection

For transient transfection experiments, cells were infected with adenoviruses encoding dominant negative p38 MAPK (Ad-Dn-p38), wild type of p38 MAPK (Ad-Wt-p38), constitutively active MKK3/6 (Ad-Ca-MKK3/6), kinase-deficient ILK (Ad-Kd-ILK), or wild type ILK (Ad-Wt-ILK) with multiplicity of infection of 100 (MOI = 100). Following overnight incubation at 37°C, SMCs were replenished with fresh medium. Transfection efficiency was measured by fluorescence microscopy and Western blot analysis at 36 hours post transfection. For all experiments, transfected SMCs were used at 36 hours post transfection. For ILK RNA inhibition experiments, SMCs were transfected with 20 nM of a 21-base pair double-stranded small interfering RNA (siRNA) molecule targeting the PH domain of ILK or a control nonspecific siRNA. ILK siRNA was introduced to cells using 2.5 μl of siLentFect Transfection Reagent according to the manufacturer's protocol (Bio-Rad Laboratories, Mississauga, ON, Canada). Twenty four hours post-transfection cells were replenished with fresh serum-containing medium, incubated for 96 hours, and then used for various experiments. ILK silencing was determined by Western blot of transfected lysates with an anti-ILK antibody.

### Western Blot Analysis

Cells either untreated or treated with various experimental reagents were washed twice with ice-cold PBS, and kept on ice for 15 min in lysis buffer containing 50 mM pyrophosphate, 50 mM, NaF, 50 mM NaCl, 5 mM EDTA, 5 mM EGTA, 100 μM Na_3_VO_4_, 10 mM HEPES (pH 7.4), 0.1% Triton X-100, 10 μg/ml leupeptin, and 1 mM phenylmethylsulfonyl fluoride. Cell lysates were collected by scraping and protein concentration was determined using Bradford assay. Extracted protein (40-80 μg) was fractionated by electrophoresis in 7% to 9% sodium dodecyl sulfate-polyacrylamide gels, transferred to nitrocellulose membranes, and blocked with PBS containing 0.1% Tween-20 and 5% non-fat dry milk for 1 hour. Afterward, the membrane was incubated with specific primary antibody overnight at 4°C, followed by secondary antibody for one hour at room temperature. Immunoblots were visualized with an enhanced chemiluminescence detection system according to the protocol of the manufacturer (Pierce Biotechnology, Rockford, IL, USA). Densitometry analysis was performed by using the National Institutes of Health ImageJ software (version 1.27z). Density values for proteins were normalized to the level for control groups (arbitrarily set to 1.0-fold).

### ILK Kinase Assay

Kinase assay was performed using 250 μg of protein lysates immunoprecipitated with 5 μg ILK antibody and protein A Sepharose beads overnight at 4°C, while shaking. The immunocomplex was washed twice with high salt NP-40 lysis buffer (containing 750 mM NaCl), and then three times with wash buffer containing 50 mM HEPES pH 7.5, 85 mM KCl, 10 mM ethyleneglycol tetraacetate (EGTA), 0.1% Tween 80, 1 mM Na_3_VO_4_, 10 mM Mg_2_Cl. The kinase assay was performed in the reaction buffer containing 0.5 μg ATP (250 μM ATP and 5 μCi [γ -^32^P] ATP) and 5 μg of myelin basic protein (MBP) as substrate at 30°C for 20 minutes in a shaker-incubator. Samples were fractionated by electrophoresis in a 12% sodium dodecyl sulfate-polyacrylamide gel, and phosphorylation of substrate by ILK was measured by a scintillation counter autoradiography.

### Cell Migration Assay

Cell migration was measured using QCM™ Transwell Colorimetric Cell Migration Assay according to the manufacturer's protocol (Chemicon International, Temecula, CA, USA). Briefly, 1 × 10^5 ^serum-starved SMCs were loaded onto the upper well of the chamber in the presence or absence of inhibitors while lower wells were filled with culture medium with or without PDGF-BB (25 ng/ml). Following 12 hours incubation, non-migrating cells on the upper side of membranes were removed by wiping and rinsing, and migrated cells were counted using colorimetric assay. Data are represented as fold change in cell migration where migratory rates for control groups are arbitrarily set to 1.0 fold.

### Wound Healing Assay

Sub-confluent transfected or non-transfected SMCs were grown on glass coverslips. Following overnight serum starvation, cell cultures were scratched with a sterile pipette tip to form a wound, washed with pre-warmed sterile PBS, and incubated with medium in the presence or absence of inhibitors for 18-24 hours. At desired timepoints post injury cells were fixed and subjected to imaging using Nikon inverted microscope and Spot digital camera.

### Cell Viability Assay

Sub-confluent SMCs were plated in a 24-well culture plate and treated with various doses of PDGF for 24 hours. The CellTiter 96^® ^AQ_ueous _Non-Radioactive Cell Proliferation Assay (MTS) was used to measure cell viability according to the manufacturer's protocol (Promega, Madison, WI).

### Actin Cytoskeleton Staining

Cells were grown on glass slides and subjected to various treatments. At desired timepoints cells were fixed (3.7% formaldehyde for 15 min & 70% ethanol for 2 min). Following permeabilization in 0.1% Triton X-100, cells were incubated with AlexaFluor 488-labelled phalloidin (Molecular Probes, Invitrogen Detection Technologies) for 1 hour in room temparature. Glass slides were mounted and sealed using ProLong^® ^Gold anti-fade reagent (Molecular Probes, Invitrogen Detection Technologies), and then imaged with Olmpus inverted microscope and Spot digital camera.

### Statistical Analysis

Two-way analysis of variance (ANOVA) with multiple comparisons andpaired Student's t tests were performed. Values shown are the mean ± standard deviation. A value of *P *< 0.05 was considered significant.

## Competing interests

The authors declare that they have no competing interests.

## Authors' contributions

The scientific idea and experimental design were developed by ME and CVB. Experiments were performed by ME, SAY. ILK kinase assay was performed by VG. Manuscript was written by ME and was revised by CVB and SD. All authors read and approved the final manuscript.

## Supplementary Material

Additional file 1 **Figure 1S. Effect of PDGF treatment on mouse aortic SMCs migration and proliferation**. Mouse SMCs were treated with increasing doses of PDGF-BB for 24 hours and cell migration and proliferation were measured. Cells treated with 10% serum were used as the positive control. As shown, 25 ng/ml of PDGF-BB significantly increased SMCs migration (3 fold increase) with no effect on cell proliferation indicating that the observed increase in cell migration is not due to the proliferatory effect of PDGF-BB in SMC culture. Data is representative of three independent experiments (n = 3 of each culture condition). Click here for file

Additional file 2 **Figure 2S. Kinetics of MAPKs activation in mouse aortic SMC culture**. PDGF-BB treatment resulted in phosphorylation and activation of all three members of MAPKs family in mouse aortic SMCs. Data represents three independent experiments. Click here for file

Additional file 3 **Figure 3S. PDGF induces LIMK and cofilin phosphorylation in mouse aortic SMCs**. Aortic SMCs were serum starved overnight and then treated with 25 ng/ml of PDGF-BB. Cell lysates were collected in various timepoints and phosphorylation of LIMK and cofilin was measured. Data represents three independent experiments. Click here for file
