# Supplementary figures and images for: Integrin-linked kinase functions as a downstream signal of platelet-derived growth factor to regulate actin polymerization and vascular smooth muscle cell migration
Source: BMC Cell Biol. 2010 Feb 23;11:16. doi: 10.1186/1471-2121-11-16 (PMC2838830; doi:10.1186/1471-2121-11-16)

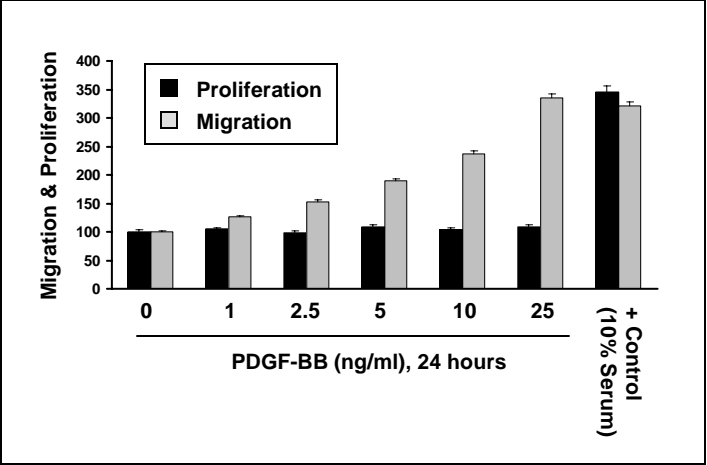

Supplement: Additional file 1 — Figure 1S. Effect of PDGF treatment on mouse aortic SMCs migration and proliferation. Mouse SMCs were treated with increasing doses of PDGF-BB for 24 hours and cell migration and proliferation were measured. Cells treated with 10% serum were used as the positive control. As shown, 25 ng/ml of PDGF-BB significantly increased SMCs migration (3 fold increase) with no effect on cell proliferation indicating that the observed increase in cell migration is not due to the proliferatory effect of PDGF-BB in SMC culture. Data is representative of three independent experiments (n = 3 of each culture condition). [file 1471-2121-11-16-S1.PDF]

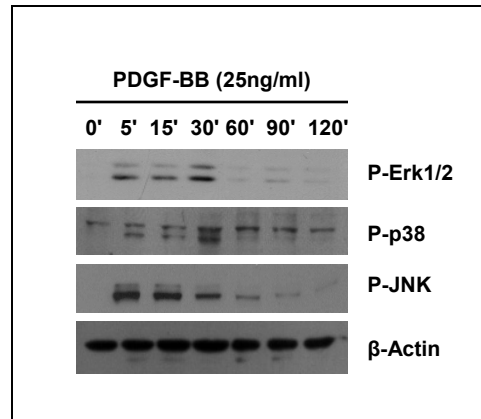

Supplement: Additional file 2 — Figure 2S. Kinetics of MAPKs activation in mouse aortic SMC culture. PDGF-BB treatment resulted in phosphorylation and activation of all three members of MAPKs family in mouse aortic SMCs. Data represents three independent experiments. [file 1471-2121-11-16-S2.PDF]

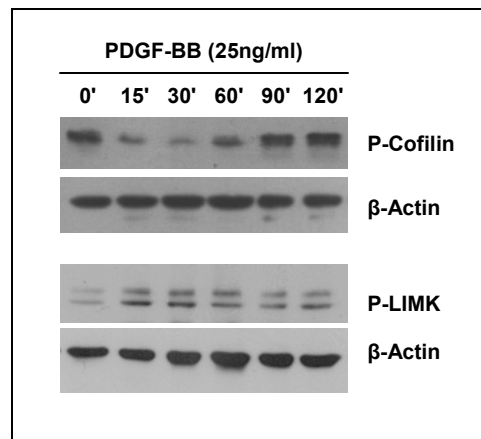

Supplement: Additional file 3 — Figure 3S. PDGF induces LIMK and cofilin phosphorylation in mouse aortic SMCs. Aortic SMCs were serum starved overnight and then treated with 25 ng/ml of PDGF-BB. Cell lysates were collected in various timepoints and phosphorylation of LIMK and cofilin was measured. Data represents three independent experiments. [file 1471-2121-11-16-S3.PDF]
